# A miRNA expression signature that separates between normal and malignant prostate tissues

**DOI:** 10.1186/1475-2867-11-14

**Published:** 2011-05-27

**Authors:** Jessica Carlsson, Sabina Davidsson, Gisela Helenius, Mats Karlsson, Zelmina Lubovac, Ove Andrén, Björn Olsson, Karin Klinga-Levan

**Affiliations:** 1 Systems Biology Research Centre - Tumor Biology, School of Life Sciences, University of Skövde, Skövde, Sweden; 2 Systems Biology Research Centre - Bioinformatics, School of Life Sciences, University of Skövde, Skövde, Sweden; 3 Department of Laboratory Medicine, Örebro University Hospital, Örebro, Sweden; 4 Department of Urology, Örebro University Hospital, Örebro, Sweden; 5 School of Health and Medical sciences, Örebro University, Örebro, Sweden

## Abstract

**Background:**

MicroRNAs (miRNAs) constitute a class of small non-coding RNAs that post-transcriptionally regulate genes involved in several key biological processes and thus are involved in various diseases, including cancer. In this study we aimed to identify a miRNA expression signature that could be used to separate between normal and malignant prostate tissues.

**Results:**

Nine miRNAs were found to be differentially expressed (*p *<0.00001). With the exception of two samples, this expression signature could be used to separate between the normal and malignant tissues. A cross-validation procedure confirmed the generality of this expression signature. We also identified 16 miRNAs that possibly could be used as a complement to current methods for grading of prostate tumor tissues.

**Conclusions:**

We found an expression signature based on nine differentially expressed miRNAs that with high accuracy (85%) could classify the normal and malignant prostate tissues in patients from the Swedish Watchful Waiting cohort. The results show that there are significant differences in miRNA expression between normal and malignant prostate tissue, indicating that these small RNA molecules might be important in the biogenesis of prostate cancer and potentially useful for clinical diagnosis of the disease.

## Background

Prostate cancer is the most common type of cancer in men and accounted for 36% of all male cancer cases in Sweden during 2009. This type of cancer is a heterogeneous disease where some men have an aggressive lapse, while others have a slower development [1]. During the last years, prostate specific antigen (PSA) has been used as a biological marker for this disease. However, since the false positive rate for PSA values is very high, there is an urgent need for new and improved markers [2].

The first microRNA (miRNA) was discovered in 1993 by Ambros and colleagues while they were performing a genetic screen in *Caenorhabditis elegans*. They identified a gene, later named *lin-4*, which does not code for a protein but rather for a 22 nucleotide long RNA molecule. It was shown that the function of this small RNA is to repress the expression of the mRNA *lin-14*, by binding to the 3'UTR of the gene [3]. Later it was discovered that miRNAs is a class of small RNAs (18-24 nt), that regulate gene expression post-transcriptionally and they have been found in plants, animals and DNA viruses [4-9]. MicroRNAs play a key role in the regulation of genes involved a diverse range of biological processes including development, cell proliferation, differentiation and apoptosis [10,11]. Approximately 1048 human miRNAs have been identified to date (miRBase release 16) [12] and it is believed that miRNAs regulate about 30% of all protein coding human genes [13,14].

Since many miRNAs are differentially expressed between normal and malignant tissues, as shown in e.g. breast and pancreatic cancer, miRNA expression profiles have potential as tools for diagnosis and prognosis of cancer [15-20]. It has been shown that expression profiles of miRNAs could be used to classify and correctly diagnose even poorly differentiated tumor samples with higher accuracy than mRNAs. Lu *et al.*, investigated tumors with histologically uncertain cellular origin for which a clinical diagnosis was established by anatomical context (colon, ovary, lung, breast and diffuse large B cell lymphoma) and showed that miRNA expression profiles could classify 12 out of 17 samples correctly while, when using mRNA expression, only one out of 17 samples was correctly classified. In addition, biomarker sets consisting of just a few miRNAs were informative enough to differentiate between tissue types [16].

Several attempts to find a miRNA expression profile for prostate cancer has been made during the last years but the results have been inconclusive. At present there are many conflicting results in the literature where results often segregate between different data sets, which can be due to study design, sample collection methods or the sensitivity and specificity of the different platforms used. Even though the results are conflicting, several studies indicate that it is possible to find a miRNA expression signature that can separate between normal and malignant prostate tissues [19,21-26].

In this study we aimed to identify a diagnostic miRNA expression signature, i.e. a set of miRNAs with expression profiles that consistently differ between normal and malignant prostate tissues. If such an expression signature can be identified and shown to have high classification accuracy, then it can potentially serve as the basis for a future diagnostic tool for prostate cancer.

## Results

In this study we included malignant prostate tissue and adjacent normal prostate tissue from twenty patients of the Swedish Watchful Waiting cohort, which consists of men with localized prostate cancer diagnosed by transurethral resection of the prostate (Table 1). The expression of 667 unique miRNAs was analyzed using the TaqMan^® ^MicroRNA Array Set v2.0 from Applied Biosystems and miRNAs that were differentially expressed between the malignant and adjacent normal prostate tissues were identified by a paired Student's t-test. In total, 30 miRNAs were found to be differentially expressed at the 0.0001 significance level. When a more stringent *p*-value of 0.00001 was applied, a subset of nine differentially expressed miRNAs was identified. When the Benjamini-Hochberg correction was performed on the *p*-values, the differential expression of all the nine miRNAs was still significant at the *p *<0.001 level (Table 2).

**Table 1:** Patient material

Sample	Age	Gleason score	%	WHO
1	80	7	20	2
2	84	6	15	2
3	79	6	60	2
4	83	7	60	2
5	83	6	15	1
6	74	6	5	2
7	86	6	2	3
8	69	7	60	2
9	85	6	10	2
10	78	7	20	2
11	76	8	70	3
12	73	7	80	2
13	75	10	40	3
14	71	9	20	2
15	74	7	15	3
16	79	10	40	3
17	71	9	60	3
18	78	9	60	3
19	85	7	20	3
20	91	9	80	3

Sample stands for the code of the patient (ranging from 1 to 20) and the Gleason score is the total Gleason score from two different tumor areas in the same patient. WHO shows the tumor grading according to the WHO classification scale and % the percentage of tumor tissue.

**Table 2:** Differentially expressed miRNAs

miRNA	+/-	p-value	Reported no. of times	Adjusted p-value
MIR126*	**-**	**7.68e-07**	**11**	**4.50e-04**
MIR34A*	-	**1.19e-06**	**13**	**6.09e-04**
**MIR622**	+	**1.52e-06**	**9**	**3.88e-04**
**MIR195**	**-**	**3.06e-06**	**7**	**8.55e-04**
**MIR26A**	**-**	**4.05e-06**	**6**	**4.50e-04**
**MIR30D**	**+**	**4.35e-06**	**9**	**4.50e-04**
MIR29A*	-	**4.48e-06**	**4**	**6.09e-04**
MIR425*	+	**5.65e-06**	**6**	**4.50e-04**
**MIR342-3P**	+	**6.11e-06**	**4**	**6.09e-04**
MIR345	-	1.07e-05	2	1.77e-03
MIR24	-	1.08e-05	3	6.09e-04
MIR484	-	1.12e-05	2	1.16e-03
MIR497	+	1.13e-05	4	6.09e-04
MIR30E	+	1.22e-05	3	6.09e-04
MIR93	+	1.29e-05	3	1.64e-03
MIR221*	-	1.37e-05	1	1.64e-03
MIR774	+	1.44e-05	2	1.64e-04
MIR152	-	2.34e-05	0	1.37e-03
MIR27B	-	2.47e-05	4	8.62e-04
MIR101*	+	3.34e-05	2	4.69e-04
MIR145*	-	3.51e-05	1	4.69e-04
MIR143	-	4.65e-05	1	1.40e-03
MIR100	-	5.12e-05	1	6.09e-04
MIR519B-3P	+	5.12e-05	2	1.40e-03
MIR200C*	+	6.66e-05	1	3.88e-04
MIR501-5P	+	6.80e-05	0	1.12e-03
MIR200C	+	6.84e-05	2	3.88e-04
MIR30A	+	6.84e-05	0	1.64e-03
MIR191	+	7.85e-05	1	6.09e-04
MIR30B	-	9.43e-05	1	2.06e-03

MicroRNAs differentially expressed between normal and malignant tissue samples of the prostate with *p <*0.0001. The nine miRNAs in bold text were also significant at the 0.00001 level. The signs +/- indicate if the miRNAs were up- or down-regulated in the malignant tissue compared to the adjacent normal prostate tissue. The reported number of times stands for the number of repetitions in which the miRNA was reported as differentially expressed in the 15-fold cross-validation test.

**Figure 1:**
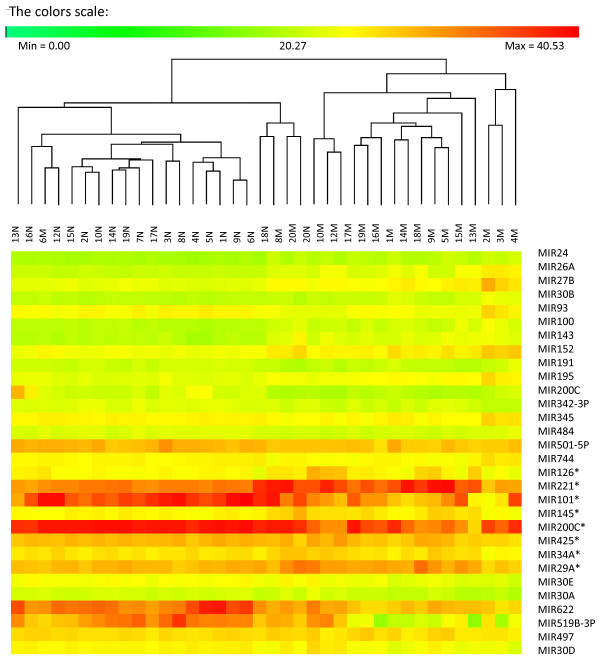
**miRNA expression signature consisting of 30 differentially expressed miRNAs**. Differentially expressed miRNAs (*p *<0.0001) were clustered and the results show that the expression profiles of these 30 miRNAs could be used to separate the normal (N) from the malignant (M) tissue samples with the exception for three malignant samples (6M, 8M and 20M).

**Figure 2:**
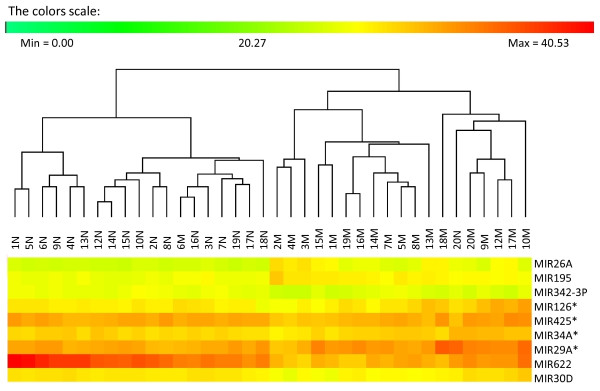
**miRNA expression signature consisting of nine differentially expressed miRNAs**. Differentially expressed miRNAs (*p *<0.00001) were clustered and the results show that the expression profiles of these nine miRNAs could be used to separate between the normal (N) and malignant (M) tissue samples with the exception of one normal sample (20N) and one malignant sample (6M).

**Figure 3:**
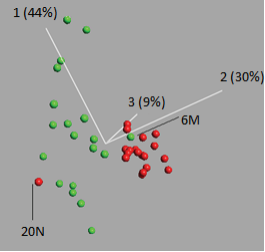
**Principal component analysis of the nine miRNA expression signature**. Principal component analysis of the nine miRNAs differentially expressed at the 0.00001 significance level. Only two samples (20N and 6M) were misplaced. The percentages on the axes describe the amount of variance that is picked up by the principal components in that direction. Green: malignant tissue sample, red: normal tissue sample.

Hierarchical clustering of the two sets of differentially expressed miRNAs was performed, showing that both these miRNA expression signatures could be used to separate between the normal and malignant prostate tissues, with the exception of three and two misplaced samples, respectively (Figure 1, Figure 2). The PCA analysis performed on the smaller expression signature, including nine miRNAs, confirmed the results from the hierarchical clustering (Figure 3).

**Figure 4:**
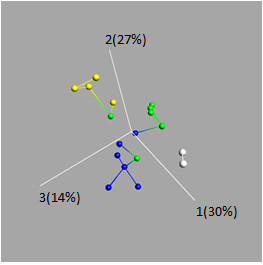
**Principal component analysis to find subgroups within the malignant samples**. A set of 16 miRNAs could be used to separate the malignant samples according to Gleason scores (GS) with the exception of three samples: one GS 6 sample placed in the GS 7 group, one GS 7 sample placed in the GS 6 group, and one GS 7 sample placed in the GS 9 group. In the figure, one GS 7 sample is hidden behind another GS 7 sample. The strings between samples correspond to their nearest neighbors. Blue: GS 6, green: GS 7, yellow: GS 9, white: GS 10.

A PCA analysis of all 667 unique miRNAs was performed to find subgroups among the 19 malignant samples included in the analyses. By gradually decreasing the *p*-value until groups emerged, we found a threshold value (*p *<0.017) that could be used to identify a set of 16 miRNAs, which arranged the samples into four groups. We then evaluated these groups for correspondence with Gleason scores and found that, with the exception of three samples, the groups represented the four Gleason scores included in the study (Figure 4).

**Figure 5:**
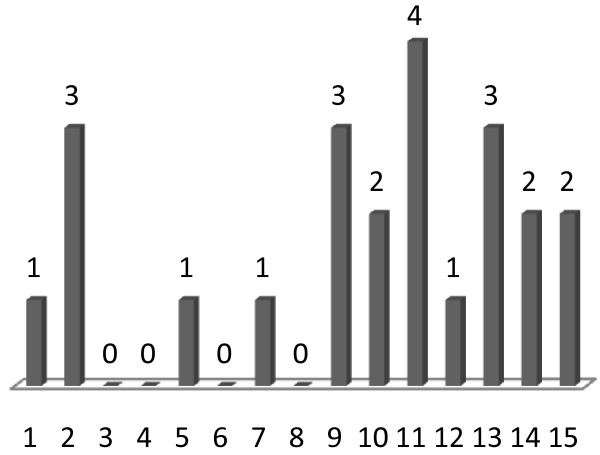
**Number of misclassified samples in cross-validation procedure**. Number of misclassified samples in each of the 15 repetitions performed in the generalization test. The error rate of the clusterings range from 0 to 40%, with an average error rate of 15.3%, meaning that on average, 1.5 of the 10 test samples was placed in a cluster where the majority of the samples belonged to the opposite class (malignant or normal).

To analyze whether the clear separation between the normal and malignant tissues was due to the fact that the same sample set was used for deriving the expression signature and for testing its performance, we performed a generalization test using cross-validation. We randomly chose 28 samples (14 malignant and the 14 corresponding normal samples) and used them for identification of differentially expressed miRNAs (*p *<0.0001). The expression signature of these miRNAs was then used to cluster the remaining 10 samples. This selection and clustering procedure was repeated 15 times (15-fold cross-validation). In each repetition we evaluated the hierarchical clustering by setting a threshold resulting in three clusters, since we had one outlier in many of the clusterings. A perfect separation between the normal and malignant tissues was found in four of the repetitions. In four cases, one sample was misplaced (error rate 10%), which means that one normal sample was placed in a cluster where the majority of samples were malignant, or vice versa. In three cases, two samples were misplaced, in three cases, three samples were misplaced, and in one case, four samples were misplaced (Figure 5). Thus, the average error rate was 15%. We also analyzed how many times the nine most significant miRNAs were identified as differentially expressed in the 15 repetitions of the generalization test, and found that this number ranged from four to 13 (Table 2). A permutation test was also performed, where the cross-validation procedure was repeated with randomly permuted class labels, but this did not produce any meaningful clusterings that could be interpreted in terms of classification accuracy.

## Discussion

In the present study, we aimed to find miRNAs with expression profiles that consistently differ between normal and malignant prostate tissues. We randomly selected 20 cases from the well-defined Swedish Watchful Waiting cohort (Table 1) and investigated the miRNA expression in malignant and the adjacent normal tissue in each individual in order to get a matched control for each case. 19 samples were used in the subsequent analyses since one sample had to be excluded due to a technical error in handling the qPCR data.

Differentially expressed miRNAs were detected by applying a paired Student's t-test. The test revealed that 30 miRNAs were differentially expressed at a *p *< 0.0001 significance level and nine miRNAs at a more stringent level (*p *< 0.00001). When a paired Wilcoxon test was applied to the data, 18 miRNAs were identified as differentially expressed (*p *< 0.0001) between the normal and malignant samples. All except five of these miRNAs were also detected as differentially expressed by the paired Student's t-test and seven of the most differentially expressed from the t-test were also detected as differentially expressed by the Wilcoxon test (See Additional file 1). Due to the largely overlapping results, we decided to proceed with the results from the t-test under the assumption that the data used in the study is approximately normally distributed.

The two sets consisting of nine and 30 differentially expressed miRNAs were further analyzed in order to find out if they could be used as expression signatures to correctly separate between the normal and malignant tissues. Hierarchical clustering was performed on the larger expression signature, including 30 miRNAs, and the analysis revealed that 16 out of 19 malignant samples were correctly classified, as well as all of the normal samples (Figure 1). The hierarchical clustering analysis was also applied for the smaller expression signature consisting of nine miRNAs. Using this expression signature, 18 of the 19 malignant samples and 18 of the 19 normal samples were correctly classified. The reason for one of the malignant samples being misclassified was probably that it belongs to a low grade tumor (Gleason score of 6). Its miRNA expression pattern may therefore be more similar to normal tissues than to the fully transformed malignant tumors with a higher Gleason scores. However, all other GS 6 malignant samples were correctly classified. The malignant sample that was misclassified had a low percentage of tumor cells (5%), which also might explain why it was placed in the normal cluster. On the other hand, the sample containing only 2% of tumor cells (7M) was correctly classified within the malignant cluster, which may indicate in some cases, the expression signature also works for tissues with a low percentage of tumor cells. A normal sample was also misclassified (20N), and we hypothesize that this might be due to that the normal tissue surrounding the tumor area might have been affected by the tumor, a phenomena called TINT (tumor indicating normal tissue) that has begun to be discussed within the prostate cancer area recently [27]. The PCA analysis of the nine differentially expressed miRNAs confirmed the results from the clustering analysis, as the same samples (6M and 20N) were misclassified using this test (Figure 2, Figure 3).

A miRNA expression signature that will be used for clinical purposes should include a limited number of miRNAs due to practical and economic reasons. Thus, in addition to our initial set of 30 differentially expressed miRNAs we also analyzed a reduced expression signature including the nine most differentially expressed miRNA genes. We found that the smaller expression signature could classify normal and malignant samples more correctly than the larger signature, which is probably due to the more stringent approach when detecting differentially expressed miRNAs (a lower *p*-value).

Six of the miRNAs (MIR26A, MIR126*, MIR195, MIR30D, MIR29A* and MIR342-3P) included in the smaller expression signature, have previously been described to be involved in the development of prostate cancer [19,22-25,28,29]. The expression of MIR126* has been investigated in prostate cancer in two previous studies and the results from these studies correspond well with our results as the miRNA was downregulated in both studies [23,29]. Porkka *et al.*, investigated the expression of three of the miRNAs included in our expression profile, MIR195, MIR26A and MIR29A* [22]. Their results correspond well with the results from our study since we found that all these three miRNAs were downregulated in malignant prostate tissues. MIR26A and MIR30D have also been shown to be downregulated in malignant prostate tissue in a another study [24] while in two other studies MIR26A together with MIR195 were found to be upregulated in malignant prostate tissues [19,25]. The results from the previous studies validate our results that MIR126*, MIR195, MIR26A, MIR29A* and MIR30D are differentially expressed in prostate cancer.

Three of the miRNAs in the signature (MIR26A, MIR126* and MIR34A*) have experimentally validated target genes (Table 3). *SLC45A3*, a target gene of MIR126* encodes a prostate specific antigen called prostein [29]. There are five validated target genes of MIR26A, *SMAD1, PLAG1, TGFBR2, SERBP1 *and *EZH2*, and one validated target gene, *NOTCH*1, of MIR34A* [19,30-35]. These target genes are involved in pathways related to e.g. cell growth and proliferation. None of the miRNAs in the small expression signature seems to be prostate specific and many of them are differentially expressed in several other diseases, such as lung cancer and leukemias [36-41], which indicates that these miRNAs might be important in general cancer development. This means that the expression profile from a single miRNA within this nine miRNA expression signature may not be reliable for diagnosis of prostate cancer specifically. However, the combination of the expression profiles of all nine miRNAs could potentially be prostate specific and thus be used for diagnostic purposes, even in cases where prostate samples are replaced by other cell types, for example circulating tumor cells [42,43].

**Table 3:** Validated target genes

miRNA	Validated target genes
MIR126*	SLC45A3 [29]
MIR34A*	NOTCH1 [35]
MIR622	-
MIR195	-
MIR26A	SERBP1,SMAD1,PLAG1,TGFBR2,EZH2 [19,30-34]
MIR30D	-
MIR29A*	-
MIR425*	-
MIR342-3P	-

Validated target genes for the nine most significantly differentially expressed miRNAs. Only three of the miRNAs currently have validated target genes.

- No validated target genes

In order to test the generality of the expression signature, we randomly chose 28 samples (14 malignant and the 14 corresponding normal tissues) to find a new set of differentially expressed miRNAs, which was then used to cluster the remaining 10 samples. When repeated 15 times, this analysis indicated that regardless of the selection of 28 randomly chosen samples, at least one of the nine most differentially expressed miRNAs from our first analysis was identified as differentially expressed (Table 2). We also analyzed how many times each of the nine differentially expressed miRNAs in our signature were chosen as differentially expressed within this analysis (Table 2). We saw that MIR34A* and MIR126* followed by MIR622 and MIR30D are the miRNAs that are differentially expressed in most of the repetitions performed (87%, 73%, 60% and 60% of the repetitions, respectively).

No clear tendencies for how different Gleason scores cluster together were found in the hierarchical clustering analysis when using the set of nine miRNAs. We therefore performed a PCA analysis of all 667 miRNAs to find subgroups within the malignant samples. The result from this analysis was a set of 16 miRNAs that could be used to classify the samples into four subgroups, which largely corresponded with Gleason scores, since only three of the 19 samples were misplaced. These results indicate that it may be possible to find a miRNA expression signature that can be used to aid tumor classification according to Gleason scores, which could be a useful complement to the manual classification performed by pathologists today. To obtain a more certain result regarding the correspondence between miRNA expression signatures and Gleason score, a more thorough study needs to be performed, focusing on this relationship.

## Conclusions

To conclude, we have shown that a miRNA expression signature consisting of nine miRNAs could separate between the normal and malignant prostate tissues with high accuracy. This separation seems to be achievable also on unseen samples, since a cross-validation test was performed and yielded similar results (85% of samples correctly classified). We have also showed that subgroups in the malignant data, revealed by miRNA expression profiles, show high concordance with Gleason scores. The miRNA signature proposed in this study needs to be evaluated in a larger patient material and preferably with another method, such as *in situ *hybridization. The results show that there are significant differences in miRNA expression between normal and malignant prostate, indicating that these small RNA molecules might be important in the biogenesis of prostate cancer and potentially also useful for clinical diagnosis of the disease.

## Methods

### Patient material

Patients were recruited from the population-based Swedish Watchful Waiting cohort [44], consisting of 1256 men with localized prostate cancer. These men had symptoms of benign prostatic hyperplasia (lower urinary tract symptoms) and were subsequently diagnosed with prostate cancer through transurethral resection (TUR-P). All men in this study were determined at the time of diagnosis to have clinical stage T1a or T1b, Mx, and Nx (small tumor, no metastases and no lymph node involvement), according to the staging system of the 2002 American Joint Committee on Cancer, called Classification of Malignant Tumors (TNM) [45]. The prospective follow-up time of this cohort is now up to 30 years. This study includes samples from men who were diagnosed at the University Hospital in Örebro (1977-1991) and at four centers in the southeast region of Sweden: Kalmar, Norrköping, Linköping, and Jönköping (1987-1999). The study was approved by the ethical committee in the Uppsala-Örebro region (Application number M58-05). The material consisted of formalin fixed paraffin embedded (FFPE) malignant prostate tissues from 20 cases and the adjacent normal tissue from each case, i.e. in total 40 paired samples. We collected cases randomly within each category of Gleason score (6-10) to get an equal distribution of histological differentiation between low grade (6-7) and high grade (8-10) Gleason scores. In addition, the tumor material consisted of different percentages of tumor cells in order to reflect the clinical reality (Table 1).

### MicroRNA qPCR arrays

The TaqMan^® ^MicroRNA Array Set v2.0 from Applied Biosystems was used in this study (Applied Biosystems, Foster City, CA, USA). It consists of two cards (Card A and Card B) containing 364 TaqMan^® ^MicroRNA assays plus 20 control assays per card, which enables quantification of 667 unique human miRNAs in total. Card A contains miRNAs that tend to be functionally defined, and are broadly and/or highly expressed. The miRNAs in card B are narrowly expressed and/or expressed at low levels and are usually not functionally defined.

### RNA extraction and cDNA preparation

Malignant and adjacent normal tissue areas on the paraffin blocks were marked by a pathologist prior to punching out 3-4 cores (ø 0.6 mm) using the Tissue Micro Array equipment (Pathology devices, Westminster, USA). The Recover All Total Nucleic Acid Isolation Kit optimized for FFPE samples (Ambion, Foster City, CA, USA) was used to extract total RNA. A reverse transcription reaction of 4-10 ng of total RNA was performed using the TaqMan^® ^MicroRNA reverse transcription kit and Megaplex^™ ^RT primers, human pool v2.0 (Applied Biosystems). Subsequently, the cDNA samples were pre-amplified using Megaplex^™ ^PreAmp primers and TaqMan® Preamp master mix (Applied Biosystems).

### Quantitative PCR

The pre-amplified cDNA samples were diluted in a 0.1 X TE Buffer (pH 8.0) before use in the qPCR reaction. The diluted pre-amplified cDNA was mixed with TaqMan^® ^PCR master mix II (No AmpErase UNG, Applied Biosystems) and run in a 40 cycle qPCR reaction on the TaqMan^® ^MicroRNA A and B Cards. All reactions were performed on the Applied Biosystems 7900 HT system.

### Data analysis

Raw Ct-values (Cycle threshold, i.e. the number of cycles where the amount of amplified cDNA crosses a defined threshold) were calculated using the SDS software (Applied Biosystems), applying manually selected thresholds for each miRNA (see Additional file 2). Due to a technical error in the handling of qPCR data, one sample (sample 11) had to be excluded from further analyses.

All statistical analyses were performed in the programming software R [46]. The raw Ct values were normalized using qPCRNorm quantile normalization, which is a data-driven normalization strategy for high-throughput qPCR data [47]. To select miRNAs to be included in the expression signature, differentially expressed miRNAs were detected by applying a paired Student's t-test on the normalized data (*p *<0.0001 and *p *<0.00001) and a Benjamini Hochberg multiple testing correction (included in the multtest R package) was applied to the *p*-values. A paired Wilcoxon test was also applied for comparison (see Additional file 1).

To test the accuracy of the miRNA expression signatures, hierarchical clustering analysis was performed on the differentially expressed miRNAs using the PermutMatrix clustering tool [48]. For measurement of similarity between expression profiles, Euclidean distance was applied. Clustering was done using the average linkage rule, which means that the distance between two clusters is represented by the average of all pairwise distances between the objects contained in the two clusters.

**Figure 6:**
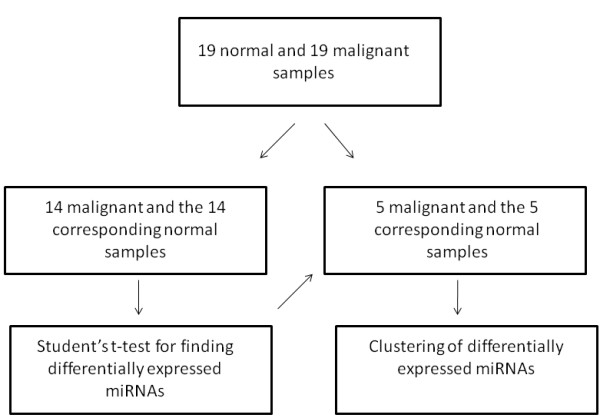
**Cross-validation procedure**. Overview over the cross-validation procedure performed to test the generality of the clustering results.

To analyze the generality of the miRNA expression signature, the following cross-validation test of generalization was applied. A set of 14 paired samples (malignant and normal from the same case) was randomly selected and a new classification signature generated by identifying the differentially expressed miRNAs on these 28 samples using a paired Student's t-test (*p *<0.0001). The remaining 10 samples (five normal and five malignant) were then clustered based on the expression values of the selected miRNAs, and the separation between normal and malignant samples was recorded. This procedure was repeated 15 times and the average classification accuracy of the 15 clusterings was calculated (Figure 6).

A principal component analysis (PCA) was performed on the miRNA expression signatures, using Omics Explorer, Version 2.0 Beta (Qlucore AB, Lund, Sweden), to confirm the results from the hierarchical clusterings. An unsupervised PCA analysis was also performed to find subgroups within the malignant tissues. In this analysis, all miRNAs were initially used and the *p-*value was gradually decreased until subgroups within the data were revealed. These groups were then evaluated for correspondence with the *a priori *known groups, i.e. normal versus malignant and the different Gleason scores.

## List of abbreviations used

cDNA: complementary DNA; DNA: deoxyribonucleic acid; miRNA: microRNA; mRNA: messenger RNA; nt: nucleotide; PCA: principal component analysis; qPCR: quantitative polymerase chain reaction; RNA: ribonucleic acid; TE: Tris-EDTA; UTR: untranslated region

## Competing interests

The authors declare that they have no competing interests.

## Authors' contributions

JC carried out the laboratory work, performed all analyses and drafted the manuscript. SD participated in the laboratory work. GH participated in the design of the study and the laboratory work and supervised the project. MK carried out the pathological marking of the tissues and supervised the project. ZL supervised the project and helped drafted the manuscript. OA, BO and KKL participated in the design of the study, supervised the project and helped drafted the manuscript. All authors read and approved the final manuscript.

## Supplementary Material

Additional file 1 **Paired Wilcoxon test results**. Differentially expressed miRNAs according to the paired Wilcoxon test performed. A plus sign means that the miRNA also was identified in the paired Student's t-test while a negative sign means that the Student's t-test did not detect this miRNA as differentially expressed (*p *<0.0001) (PDF-file). Click here for file

Additional file 2 **microRNA qPCR array raw data**. Raw data from microRNA qPCR card A and B (XLS-format). Click here for file
