# Supplementary material for: A miRNA expression signature that separates between normal and malignant prostate tissues
Source: Cancer Cell Int. 2011 May 27;11:14. doi: 10.1186/1475-2867-11-14 (PMC3123620; doi:10.1186/1475-2867-11-14)
Supplement: Additional file 1 — Paired Wilcoxon test results. Differentially expressed miRNAs according to the paired Wilcoxon test performed. A plus sign means that the miRNA also was identified in the paired Student's t-test while a negative sign means that the Student's t-test did not detect this miRNA as differentially expressed (p <0.0001) (PDF-file). [file 1475-2867-11-14-S1.PDF]

miRNA	Students T-test
MIR26A	+
MIR93	+
MIR195	+
MIR200C	+
MIR296-5P	-
MIR343-3P	+
MIR455-5P	-
MIR501-5P	+
MIR744	+
MIR154*	+
MIR380*	-
MIR340*	-
MIR425*	+
MIR34A*	+
MIR29A*	+
MIR622	+
MIR497	+
MIR519B-3P	+

+ Detected in the T-test

- Not detected in the T-test
